# Value-Regularized Reinforcement Learning for Model Predictive Control of Autonomous Mobile Robots Under Stochastic Disturbances

**DOI:** 10.3390/s26144620

**Published:** 2026-07-21

**Authors:** Changyuan Yu, Weiguo Zhang, Qi Li, Yongdong Cheng, Zhanming Li

**Affiliations:** Xi’an Institute of Applied Optics, Xi’an 710065, China; yuchangyuan0108@163.com (C.Y.);

**Keywords:** model predictive control, reinforcement learning, robust cost design, online learning, expected Lyapunov drift, autonomous mobile robots, stochastic disturbances

## Abstract

Autonomous mobile robots depend on sensing and state estimation to provide feedback, while their controllers must remain effective under process disturbances and model mismatch. Reinforcement learning-based model predictive control (RL-MPC) learns the terminal cost online, while the deterministic RLMPC baseline uses a nominal stage cost. This study considers the control layer and assumes that the robot state is available; measurement noise and state-estimation errors are outside the modeled disturbance channel. Isotropic state regularization is introduced into the RL-MPC stage cost, yielding value-regularized RLMPC (VR-RLMPC). The same β term changes both the physical state penalty and the *N*-step terminal-value learning target without adding online optimization variables, constraints, or sampling operations. Under explicit value-function, feasibility, domain-containment, and bounded-disturbance assumptions, a Lyapunov-drift analysis yields a conditional one-step expected-drift bound outside an explicit radius. Simulations on linear and nonlinear nonholonomic vehicle systems show empirical VFA weight-update settling-step indices (CR) that are approximately 25% lower than those of RLMPC across the nominal and five model-mismatch comparisons. Across the tested mismatch range, VR-RLMPC has a worst-case performance degradation rate of 2.8%, compared with 25–49% for conventional MPC baselines; its cost improvement also increases with task difficulty. In the tested nominal setting, VR-RLMPC also outperforms an explicit stochastic temporal-difference baseline without adding online optimization variables or sampling operations.

## 1. Introduction

Autonomous mobile robots operating under stochastic disturbances require controllers that adapt online to uncertain dynamics while satisfying state and input constraints. Model predictive control (MPC) addresses this need by solving a constrained finite-horizon optimization problem online at each time step [1]. Closed-loop stability typically relies on the design of a terminal cost F(·) and a terminal constraint set $X_{f}$ [2]: F(·) must approximate the infinite-horizon optimal value function, and $X_{f}$ must be a positively invariant set. For linear systems, F(·) can be obtained analytically from the discrete algebraic Riccati equation (DARE); for nonlinear systems, an offline computation based on local linearization around the equilibrium is required. When the plant is subject to external disturbances or model parameter deviations, a terminal design based on the nominal model may fail to capture the true long-horizon cost, leading to closed-loop performance degradation or even constraint violation.

In a physical mobile robot, the control state is typically supplied by onboard sensors and a state-estimation pipeline. This work isolates the control-layer problem and assumes that the state is available at each control step. Measurement noise, state-estimation error, and multi-sensor fusion are not included in the disturbance model or evaluated in the simulations. Recent Sensors studies have used deep reinforcement learning (DRL) for mobile-robot path following and sensor-fusion-based navigation [3,4], illustrating the broader sensing–navigation context in which autonomous mobile robots operate. The uVGS-2 framework is a recent example that combines vision-based 6-DoF pose measurements with factor-graph localization [5]. Its treatment of camera calibration, illumination, line-of-sight occlusion, and noise rejection illustrates practical sensing issues upstream of the control layer.

Robust and stochastic MPC address such uncertainty within the online optimization. Tube MPC [6] forms a feasible tube by augmenting the nominal trajectory with the Minkowski sum of a robust positively invariant set, guaranteeing recursive feasibility under bounded disturbances at the expense of conservatism. Stochastic MPC [7,8] relaxes hard constraints to chance constraints, reducing conservatism but incurring higher online computation. Recent extensions expand these frameworks: Paulson et al. [9] address joint chance-constraint feasibility, Arcari et al. [10] incorporate parametric uncertainty, Bujarbaruah et al. [11] unify adaptive robust and stochastic MPC, and Köhler et al. [12] provide a computationally efficient framework for uncertain nonlinear systems. On the theoretical side, the input-to-state stability (ISS) Lyapunov approach [13,14] establishes robust stability of MPC through value-function decrease conditions, and Allan et al. [15] further prove inherent robustness of MPC without terminal constraints.

These methods explicitly model uncertainty in the online optimization and are suitable when the uncertainty set, chance-constraint level, or invariant tube is part of the controller specification. The present study addresses a narrower question within RL-MPC: how to couple the constrained control objective and the learned terminal-value target to a disturbance-sensitive state penalty without enlarging the online MPC problem. The proposed regularization modifies the stage-cost signal that drives value-function learning while preserving the RLMPC decision variables, constraints, and sampling structure. It is a low-complexity learning-side intervention for additive process disturbances rather than a substitute for tube or stochastic MPC.

Online terminal-cost learning provides an alternative to a fixed offline design. Gaussian process (GP) modeling [16,17] learns dynamics residuals online and quantifies uncertainty, but its inference cost limits real-time applicability to large-scale systems. Related safety-oriented work imposes Lyapunov stability constraints in model-based RL [18], designs predictive safety filters [19], and surveys safety mechanisms for learning-based MPC [20]. RL and MPC have also been combined to learn terminal costs online. Gros and Zanon [21] treat MPC as a parameterized policy approximator and tune its parameters via temporal-difference (TD) errors, with subsequent work [22,23] introducing robustness and stability guarantees. Rosolia and Borrelli [24] iteratively construct safe sets and terminal costs from historical trajectories, while Soloperto et al. [25] provide closed-loop performance guarantees. These RL-MPC methods build on approximate dynamic programming (ADP) [26]; Lewis et al. [27] and Kiumarsi et al. [28] survey RL for optimal control, and Al-Tamimi et al. [29] prove convergence of the discrete-time Hamilton–Jacobi–Bellman (HJB) equation in this framework. Recent DRL–MPC applications address cloud-assisted path tracking and distributed cooperative control [30,31]; their different learning objects and evaluation protocols make them contextual references rather than direct numerical baselines for online terminal-cost learning.

Regularized RL provides a separate point of comparison. General regularized Markov decision process formulations modify the Bellman operator through a convex policy regularizer, commonly based on entropy or Kullback–Leibler divergence [32]. VR-RLMPC does not regularize the policy distribution, action entropy, or policy-update divergence. Instead, βI changes the physical quadratic state penalty inside the constrained RL-MPC problem, and this modified stage cost enters the TD target used to learn the terminal value function. The term “value-regularized” therefore denotes stage-cost shaping of online terminal-value learning rather than standard entropy- or policy-regularized RL.

Lin et al. [33] propose an RL-MPC scheme that bridges RL and MPC through policy iteration (PI): MPC serves as the policy generator, while VFA estimates the terminal cost online via TD learning; the two alternate until convergence. This scheme removes the need for an offline terminal set and terminal controller, and provides convergence and closed-loop stability guarantees. The cost function in this formulation, however, assumes a deterministic system. Under additive process disturbances, the nominal terminal-value target may not represent the disturbed finite-horizon behavior. This work examines disturbance robustness in RL-MPC through a direct modification of the stage cost. Specifically, VR-RLMPC places the same β term in the physical state penalty and the *N*-step TD target while retaining the online MPC structure. The analysis uses deterministic PI monotonicity as its baseline and treats disturbances through a conditional expected-drift bound and model-mismatch evaluation.

This work proposes value-regularized RLMPC (VR-RLMPC), which modifies the stage cost within the RL-MPC framework to couple constrained control with online terminal-value learning under process disturbances. The main contributions are as follows.

1. Isotropic state regularization is embedded in the stage cost so that the value function learning target penalizes state deviations more strongly. The same β term changes both the physical state penalty and the *N*-step TD target used for terminal-value learning, without changing the online MPC dimension, constraints, or sampling structure. This coupled control–learning modification distinguishes VR-RLMPC from changing the MPC weight alone and from policy-distribution regularization in standard regularized RL. Two effects provide a mechanism-level explanation for the observed empirical weight-update settling changes: amplification of the TD stage-cost signal and reduction of the condition number of the stage-cost matrix. Direction-dependent priorities remain encoded by *Q* and the constraint sets.
2. Under explicit VFA, Bellman-residual, feasibility, domain-containment, and bounded-disturbance assumptions, a Lyapunov-drift analysis gives a conditional one-step expected-drift bound. For fixed disturbance covariance, Hessian bound, and Bellman residual, the radius outside which the expected drift is negative decreases as β increases; this is not a recursive-feasibility or trajectory-level probability guarantee.
3. The proposed regularization is evaluated on a nonlinear nonholonomic vehicle system through ablation and model-mismatch simulations. The evaluation also includes an ${\mathrm{MPC}}_{Q_{r}}$ ablation, a stochastic-TD comparison, a matched training-budget study, and a β-sensitivity sweep. At β=0.5, empirical CR is lower in the nominal and all five model-mismatch comparisons, the cost improvement grows with task difficulty, and the observed performance degradation rate is lower than that of the conventional MPC baselines.

The remainder of this paper is organized as follows. Section 2 introduces the system model with disturbances, MPC fundamentals, and the theoretical background of value function approximation. Section 3 details the robust RL-MPC algorithm design, including the robust cost function, MPC-RL interaction procedure, convergence analysis, and the conditional expected-drift result. Section 4 presents and discusses simulation results on both linear and nonlinear systems. Section 5 concludes the paper and outlines future validation directions.

## 2. Background and System Modeling

### 2.1. System Model with Process Disturbances

Consider a discrete-time nonlinear system with additive disturbances:

(1) $$x_{k+1}=f\left(x_{k},u_{k}\right)+w_{k}$$

where $x_{k}\in R^{n}$ is the state vector, $u_{k}\in R^{m}$ is the control input, f(·) is the nominal state transition function, and $w_{k}$ denotes an external process disturbance. The nominal MPC predictions impose $x_{i|k}\in X$ and $u_{i|k}\in U$, where both sets are bounded, closed, and contain the origin. The applied input is constrained by U, but recursive satisfaction of the state constraints by the disturbed closed-loop state is neither assumed nor proved for unbounded disturbances.

**Assumption 1** (Smoothness and state availability)**.**
*$f:R^{n}\times R^{m}\to R^{n}$ is continuously differentiable with f(0,0)=0. The state $x_{k}$ is available at each time step through an idealized control interface. Measurement noise, state-estimation error, and the sensing architecture are not modeled separately. Sensor latency and estimator–controller coupling are likewise excluded. All analytical statements therefore concern the state-feedback interconnection and do not extend to an estimator–controller interconnection.*

**Assumption 2** (Simulation and analysis disturbance models)**.**
*The simulations use independent Gaussian process disturbances $w_{k}^{G}∼N\left(0,\Sigma_{G}\right)$. The expected-drift analysis uses a distinct analytical disturbance ${\tilde{w}}_{k}$ that is independent of the closed-loop history, satisfies $E[{\tilde{w}}_{k}|x_{k}]=0$ and $E\left[{\tilde{w}}_{k}{\tilde{w}}_{k}^{T}|x_{k}\right]=\Sigma_{\tilde{w}}$, and is supported on the compact set*

(2) $$W=\{\tilde{w}\;:\;∥\tilde{w}∥\;\leq \;\bar{w}\}.$$

*A symmetrically truncated Gaussian is one admissible analytical model, but its covariance is $\Sigma_{\tilde{w}}$, not automatically the covariance of the untruncated simulation noise. The theoretical result is conditional on this bounded zero-mean model and makes no recursive-feasibility, tail-risk, or trajectory-level claim for untruncated Gaussian disturbances.*

Following the deterministic RLMPC baseline, terminal-value learning is defined for the nominal disturbance-free dynamics ${\bar{x}}_{i+1}=f\left({\bar{x}}_{i},\pi \left({\bar{x}}_{i}\right)\right)$, with ${\bar{x}}_{k}=x_{k}$. The nominal infinite-horizon objective is

(3) $$J_{0}\left(x_{k},\pi \right)=\sum_{i=k}^{\infty }l\left({\bar{x}}_{i},\pi \left({\bar{x}}_{i}\right)\right)$$

where l(x,u) is the stage cost. This nominal value-function objective is distinct from the finite-horizon accumulated cost used to evaluate the persistently disturbed simulations in Section 4.1; no undiscounted infinite-horizon expected-cost claim is made for the untruncated Gaussian process.

**Assumption 3** (Positive definiteness and quadratic weights)**.**
*The nominal stage cost used in the MPC formulations is $l\left(x,u\right)=x^{T}Qx+u^{T}Ru$, where $Q=Q^{T}≻0$ and $R=R^{T}⪰0$. Consequently, l is positive definite in the state, and there exist $K_{\infty }$-class functions $\alpha_{1},\alpha_{2}$ [13] such that*

(4) $$\alpha_{1}\left(∥x∥\right)\leq \min_{u}l\left(x,u\right)\leq \alpha_{2}\left(∥x∥\right)$$

The optimal value function is defined as

(5) $$V^{*}\left(x_{k}\right)=\min_{\pi }J_{0}\left(x_{k},\pi \right)$$

with the corresponding optimal policy

(6) $$\pi^{*}\left(x_{k}\right)=\arg\min_{u_{k}}\left\{l\left(x_{k},u_{k}\right)+V^{*}\left(f\left(x_{k},u_{k}\right)\right)\right\}.$$

Assumptions 1 and 3 and the nominal objective follow the deterministic RL-MPC setting [33]; Assumption 2 separately defines the simulation disturbance and the bounded analytical disturbance used below.

### 2.2. MPC and Terminal Cost Design

MPC solves the following finite-horizon optimization at each time step *k*:

(7a) $$\begin{array}{cc} \min_{u_{0|k},\ldots ,u_{N-1|k}} & \;\sum_{i=0}^{N-1}l\left(x_{i|k},u_{i|k}\right)+F\left(x_{N|k}\right) \end{array}$$

(7b) $$\begin{array}{cc} s.t. & \;x_{0|k}=x_{k},\;x_{i+1|k}=f\left(x_{i|k},u_{i|k}\right) \end{array}$$

(7c) $$\begin{array}{cc} \; & \;x_{i|k}\in X,\;u_{i|k}\in U,\;x_{N|k}\in X_{f} \end{array}$$

where *N* is the prediction horizon, F(·) is the terminal cost, and $X_{f}$ is the terminal constraint set. Only the first input $u_{0|k}^{*}$ is applied, and the problem is re-solved at the next time step.

The terminal cost F(·) compensates the cost truncation introduced by the finite horizon, making the finite-horizon cost approximate $J_{0}$. Its design relies on linearization of the nominal model near the equilibrium. For linear-quadratic problems, $F\left(x\right)=x^{T}Px$, where *P* solves the discrete algebraic Riccati equation (DARE). For nonlinear systems, *P* is obtained from a local linearization at the equilibrium. Both approaches require an accurate model, and the terminal set $X_{f}$ must satisfy a positive invariance condition.

When the system is subject to Gaussian disturbances or model parameter deviations, the offline-computed F(·) and $X_{f}$ may fail to represent the disturbed finite-horizon closed-loop behavior. An online-learned value function approximation $\hat{V}\left(x\right)$ can directly replace F(·), bypassing the offline design.

### 2.3. Value Function Approximation and Online Policy Iteration

Given a policy π, the infinite-horizon value function under deterministic dynamics is

(8) $$V^{\pi }\left(x_{k}\right)=\sum_{i=k}^{\infty }l\left(x_{i},\pi \left(x_{i}\right)\right)$$

The optimal value function $V^{*}$ satisfies the Bellman equation (deterministic form):

(9) $$V^{*}\left(x_{k}\right)=\min_{u_{k}}\left\{l\left(x_{k},u_{k}\right)+V^{*}\left(f\left(x_{k},u_{k}\right)\right)\right\}$$

Solving (9) directly is intractable for nonlinear systems. A parameterized value function approximation (VFA) is introduced:

(10) $$\hat{V}\left(x;W\right)=W^{T}\phi \left(x\right)$$

where $\phi \left(x\right)={\left[\phi_{1}\left(x\right),\ldots ,\phi_{L}\left(x\right)\right]}^{T}\in R^{L}$ is a polynomial basis function vector and $W\in R^{L}$ is the weight vector. In the standard one-step TD form, a transition tuple $\left(x_{k},u_{k},x_{k+1}\right)$ defines the residual

(11) $$\delta_{k}=l\left(x_{k},u_{k}\right)+\hat{V}\left(x_{k+1};W\right)-\hat{V}\left(x_{k};W\right)$$

Semi-gradient updates use $\delta_{k}$ to reduce the empirical TD residual. If the learning process converges, $\hat{V}\left(\cdot ;W\right)$ approximates $V^{*}$ and serves as an online replacement for the terminal cost F(·) in (7). This one-step expression provides background notation; the reported implementation evaluates uniformly sampled states with the nominal *N*-step MPC rollouts defined in Section 3.3.

## 3. Robust RL-MPC Algorithm Design and Analysis

### 3.1. Problem Analysis

Section 2.3 introduces the value-function approximation used as the MPC terminal cost. In the reported RLMPC implementation, uniformly sampled states $x^{\left(s\right)}$ are propagated through the nominal MPC model, and the finite-horizon stage-cost sum plus the terminal VFA value forms an *N*-step policy-evaluation target. Gaussian process disturbances enter the outer closed-loop training and evaluation trajectories, but they are not sampled inside the ordinary RLMPC or VR-RLMPC terminal target. Consequently, the learner remains driven by a nominal stage-cost objective while the controller is evaluated on disturbed trajectories. VR-RLMPC modifies this nominal learning signal and the constrained control objective through the same state-weight increment βI.

### 3.2. Robust Cost Function Design

Define the modified stage cost

(12) $$l_{r}\left(x_{k},u_{k}\right)=x_{k}^{T}\left(Q+\beta I\right)x_{k}+u_{k}^{T}Ru_{k}$$

where β≥0 is the robust compensation coefficient. When β=0, this reduces to the nominal cost l(x,u).

**Remark 1** (Design motivation)**.**
*The term βI superimposes an isotropic penalty on the state weight Q, tightening the tolerance for state deviation. It also enters the terminal-value TD target through the modified stage cost, and the resulting increase in $\lambda_{\min}\left(Q+\beta I\right)$ appears in the conditional one-step expected-drift bound of Theorem 2. Under $w_{k}^{G}∼N\left(0,\sigma^{2}I\right)$, the single-step state deviation due to the disturbance is of order O(σ); the value of β should modify the learning signal without dominating the stage cost. In practice, β is chosen to be on the same order as the eigenvalues of Q. Since Q+βI⪰Q≻0, the modified cost $l_{r}$ preserves the positive-definiteness condition used in the subsequent convergence and expected-drift analyses and increases the minimum state-cost curvature.*

**Remark 2** (Scope of isotropic weighting)**.**
*The implemented state-weight matrix is $Q_{r}=Q+\beta I$, so the original directional priorities remain in Q. For the nonlinear configuration, Q=diag(1,1,0.2) becomes $Q_{r}=\mathrm{diag}\left(1+\beta ,1+\beta ,0.2+\beta \right)$, retaining a lower heading weight with reduced contrast. The model f preserves the nonholonomic coupling, while R, X, and U retain the input priorities and admissible sets. Direction-dependent regularization could encode additional actuator priorities but would require more tuning parameters; this study uses the reproducible single-parameter form.*

### 3.3. Algorithm Procedure

#### 3.3.1. MPC Stage

At each time step *k*, MPC replaces *l* with $l_{r}$ and solves the finite-horizon optimization:

(13a) $$\begin{array}{cc} \min_{u_{0|k},\ldots ,u_{N-1|k}} & \;\sum_{i=0}^{N-1}l_{r}\left(x_{i|k},u_{i|k}\right)+\hat{V}\left(x_{N|k};W_{j}\right) \end{array}$$

(13b) $$\begin{array}{cc} s.t. & \;x_{0|k}=x_{k},\;x_{i+1|k}=f\left(x_{i|k},u_{i|k}\right) \end{array}$$

(13c) $$\begin{array}{cc} \; & \;x_{i|k}\in X,\;u_{i|k}\in U \end{array}$$

where $W_{j}$ is the VFA weight after the *j*-th policy iteration. Compared with (7), problem (13) differs in two ways: (i) the stage cost changes from *l* to $l_{r}$; (ii) the terminal set constraint $X_{f}$ is removed, with the VFA terminal cost implicitly assuming the stabilization role. The MPC solver still uses the nominal dynamics f(x,u) for prediction; disturbance compensation is achieved indirectly through the βI term in $l_{r}$ and the online VFA learning. Only the first input $u_{0|k}^{*}$ is applied to the controlled plant.

#### 3.3.2. RL Stage

For each sampled state $x^{\left(s\right)}$, the nominal MPC rollout supplies an *N*-step semi-gradient target. With predicted state–input pairs ${\left\{x_{i|j}^{\left(s\right)},u_{i|j}^{*(s)}\right\}}_{i=0}^{N-1}$ and terminal state $x_{N|j}^{\left(s\right)}$, the robust TD residual is

(14) $$\delta_{j}^{\left(s\right)}=\sum_{i=0}^{N-1}l_{r}\left(x_{i|j}^{\left(s\right)},u_{i|j}^{*(s)}\right)+W_{j}^{T}\phi \left(x_{N|j}^{\left(s\right)}\right)-W_{j}^{T}\phi \left(x^{\left(s\right)}\right).$$

The corresponding unclipped batch semi-gradient update is

(15) $$W_{j+1}=W_{j}+\alpha \sum_{s=1}^{q}\delta_{j}^{\left(s\right)}\phi \left(x^{\left(s\right)}\right),$$

where α>0 is the learning rate and *q* is the number of samples per policy-iteration step. The code applies samples sequentially with $\ell_{2}$ shrinkage $\lambda_{W}$, update-norm clipping at $G_{\max}$, and elementwise projection to $\left[-W_{\max},W_{\max}\right]$. The nonlinear simulations use $\alpha =10^{-5}$, $\lambda_{W}=10^{-3}$, $G_{\max}=1$, and $W_{\max}=100$; the linear simulations use $\alpha =10^{-3}$ with the same safeguards. Learning driven by $l_{r}$ targets its nominal deterministic infinite-horizon value. A finite basis, sampled states, and finite updates provide an approximation of this target rather than exact finite-sample recovery.

**Figure 1:**
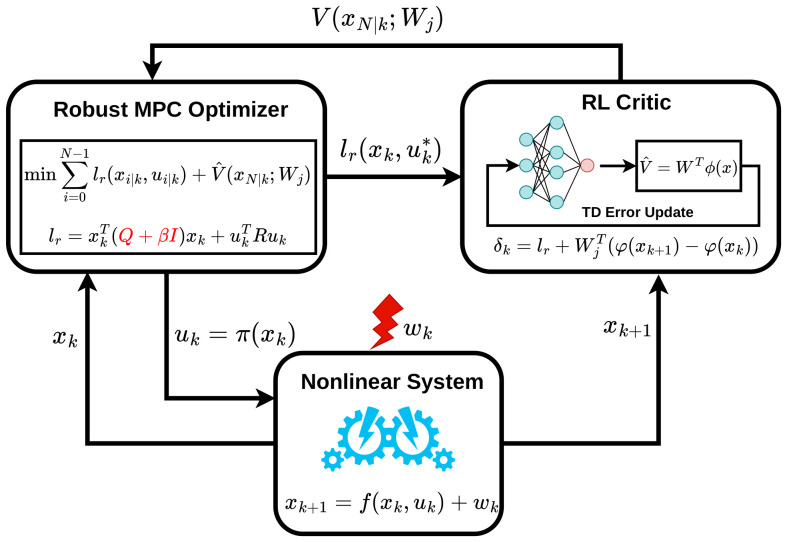
Conceptual system-level closed-loop architecture of VR-RLMPC; the implemented sampled-state nominal *N*-step VFA update is given in (14) and Algorithm 1.

Figure 1 is a system-level signal-flow schematic. The “Robust MPC Optimizer” label denotes the value-regularized constrained optimizer in (13), rather than a tube-, set-, or chance-constrained robust MPC formulation. The critic-side single-step notation is conceptual; the reported update uses the sampled-state nominal *N*-step residual in (14) and the safeguards in (15). The disturbed plant closes the state-feedback loop, whereas the sampled policy-evaluation rollouts remain nominal. The reported implementation uses the polynomial basis $\hat{V}\left(x;W_{j}\right)=W_{j}^{T}\phi \left(x\right)$, and Algorithm 1 specifies the complete procedure.
**Algorithm 1** Value-Regularized RL-MPC (VR-RLMPC)**Require:** System *f*, parameters $Q,\;R,\;\beta ,\;N,\;\alpha ,\;\epsilon ,\;q,\;\lambda_{W},\;G_{\max},\;W_{\max}$, initial weights $W_{0}$
**Ensure:** Closed-loop control policy $\mu_{W^{*}}\left(x\right)=u_{0|k}^{*}$
  1:$Q_{r}\leftarrow Q+\beta I$  2:**for** policy iteration j=0,1,2,… **do**  3:    Sample *q* states ${\left\{x^{\left(s\right)}\right\}}_{s=1}^{q}$ uniformly from X  4:    **for** s=1,…,q **do**  5:        Solve (13) with terminal weights $W_{j}$ and obtain the *N*-step nominal rollout  6:        Compute $\delta_{j}^{\left(s\right)}$ from (14)  7:        Apply the $\ell_{2}$-regularized update, clip its norm at $G_{\max}$, and project *W* to $\left[-W_{\max},W_{\max}\right]$  8:    **end for**  9:    $W_{j+1}\leftarrow$ the final projected weight after the *q* sequential updates10:    **if** $∥W_{j+1}-W_{j}∥\leq \epsilon$ **then**11:        break12:    **end if**13:**end for**14:Deployment: At each time step *k*, solve (13) with $W^{*}$ and apply $u_{0|k}^{*}$


### 3.4. Convergence Analysis

The analysis below is carried out under deterministic training ($w_{k}=0$), consistent with the analysis framework of deterministic RL-MPC. The deterministic result inherited from RLMPC concerns an idealized policy-iteration (PI) mapping in which each policy-evaluation step is completed before the policy is updated [33]. It must be distinguished from the finite-sample SGD iterates used in the implementation. In this subsection, $V_{r}^{*}$ denotes the deterministic optimal value function associated with $l_{r}$.

**Assumption 4** (Idealized policy evaluation)**.**
*For the analytical PI sequence, $w_{k}=0$, $V_{0}\left(x\right)=0$, and each outer iteration evaluates the finite-horizon Bellman operator exactly on X, *

(16) $$\begin{array}{cc} V_{j+1}\left(x\right) & =\left(T_{N}V_{j}\right)\left(x\right)\\ \; & ≜\min_{{\left\{u_{i}\right\}}_{i=0}^{N-1}}\left\{\sum_{i=0}^{N-1}l_{r}\left(x_{i},u_{i}\right)+V_{j}\left(x_{N}\right)\right\}, \end{array}$$

*subject to the nominal dynamics and constraints in* (13). *The minimum is assumed to exist for every x∈X included in the analysis.*

**Theorem 1** (Monotonicity of the idealized PI sequence)**.**
*Under Assumptions 1, 3, and 4, suppose that the nonnegative robust optimal value $V_{r}^{*}$ is a fixed point of $T_{N}$ on X. Then*

(17) $$0=V_{0}\left(x\right)\leq V_{j}\left(x\right)\leq V_{j+1}\left(x\right)\leq V_{r}^{*}\left(x\right),\;x\in X.$$

**Proof.**  Because $l_{r}\geq 0$, $T_{N}V_{0}\geq V_{0}$. The Bellman operator is order preserving: $V_{a}\leq V_{b}$ implies $T_{N}V_{a}\leq T_{N}V_{b}$. Induction therefore gives $V_{j}\leq V_{j+1}$. Likewise, $V_{0}\leq V_{r}^{*}$ and $T_{N}V_{r}^{*}=V_{r}^{*}$ imply $V_{j}\leq V_{r}^{*}$ for every *j*. □

Theorem 1 applies only to the exact PI sequence in Assumption 4. Algorithm 1 uses a finite basis, finitely many sampled states, and finite semi-gradient updates; pointwise monotonicity and a convergence rate for its weight sequence $\left\{W_{j}\right\}$ do not follow from this theorem. The simulations therefore report CR only as an empirical weight-update settling-step index rather than as verification of a finite-sample convergence theorem.

**Remark 3** (Interpretation and limit of β in the learning dynamics)**.**
*The observed weight-update settling changes can be interpreted through two effects. (i) TD stage-cost signal: the stage-cost component of the TD error* (14) *increases by ${\beta ∥x∥}^{2}$. Before empirical weight settling, this changes the scale of the effective semi-gradient signal in the SGD update* (15). *(ii) Stage-cost condition number: the condition number of $Q_{r}=Q+\beta I$ satisfies*

(18) $$\kappa \left(Q_{r}\right)=\frac{\lambda_{\max}\left(Q\right)+\beta }{\lambda_{\min}\left(Q\right)+\beta }\leq \kappa \left(Q\right)$$

*with strict inequality when β>0 and Q is not a scalar matrix. A better-conditioned stage-cost matrix reduces differences in the stage-cost weighting across state dimensions and can lower the approximation difficulty for a finite basis set ϕ. Together, these two effects provide a mechanism-level interpretation of the empirical settling-step changes observed in the nominal and mismatch comparisons. They do not constitute a convergence-rate proof. Because $\delta_{j}^{\left(s\right)}$ enters the update together with α and $\phi (x^{\left(s\right)})$, finite-sample updates can grow with larger β, learning rates, state excursions, or basis magnitudes despite the reported clipping safeguards. The present analysis does not prove bounded unclipped weights or exclude divergence for untested β–α combinations or more dynamic regimes. The sensitivity sweep instead shows that larger β can increase CR under the high-$Q_{\theta }$ configuration.*

### 3.5. Conditional Expected Lyapunov-Drift Analysis

This subsection uses a Taylor/Lyapunov decomposition [13] to derive a conditional one-step expected-drift bound for the bounded analytical disturbance in Assumption 2. The result applies to the state-feedback interconnection of Assumption 1; recursive feasibility and trajectory-level behavior require separate analysis.

**Assumption 5** (VFA Hessian boundedness)**.**
*For fixed deployed weights $W^{*}$, $V\left(x\right)≜\hat{V}\left(x;W^{*}\right)$ is twice continuously differentiable on a domain D⊇X, and there exists a constant $M_{V}>0$ such that*

(19) $$∥\nabla^{2}V\left(z\right)∥\leq M_{V},\;z\in D.$$

*For a quadratic polynomial basis, $\nabla^{2}V$ is a constant matrix and the Hessian bound holds globally; for higher-order bases, $M_{V}$ remains finite on the bounded domain used by the analysis.*

**Assumption 6** (Lyapunov upper and lower bounds)**.**
*There exist $K_{\infty }$-class functions $\underline{\alpha },\bar{\alpha }$ such that*

(20) $$\underline{\alpha }\left(∥x∥\right)\leq V\left(x\right)\leq \bar{\alpha }\left(∥x∥\right),\;\forall x\in X.$$

*These are the standard Lyapunov upper and lower bound conditions used in the ISS framework [13].*

**Assumption 7** (Nominal Bellman residual)**.**
*For the deployed MPC policy $\mu_{W^{*}}$, there is a uniform constant $\epsilon_{B}\geq 0$ satisfying*

(21) $$V\left(x\right)-V\left(f\left(x,\mu_{W^{*}}\left(x\right)\right)\right)\geq l_{r}\left(x,\mu_{W^{*}}\left(x\right)\right)-\epsilon_{B},\;x\in X.$$

*This residual bound is an explicit analytical assumption; it is not implied by Theorem 1 or by a one-step concentration inequality. The present simulations do not certify a uniform numerical value of $\epsilon_{B}$.*

**Assumption 8** (Feasibility and domain containment)**.**
*At every state to which the result is applied, problem* (13) *is feasible and returns $\mu_{W^{*}}\left(x\right)$. Moreover, for every $\tilde{w}\in W$ and t∈[0,1],*

(22) $$f\left(x,\mu_{W^{*}}\left(x\right)\right)+t\tilde{w}\in D.$$

**Theorem 2** (Conditional expected Lyapunov drift)**.**
*Under Assumptions 2, 3, and 5–8, consider*

(23) $$x_{k+1}=f\left(x_{k},\mu_{W^{*}}\left(x_{k}\right)\right)+{\tilde{w}}_{k}.$$

*For every $x_{k}\in X$ at which Assumption 8 holds,*

(24) $$\begin{array}{cc} \; & E\left[V\left(x_{k+1}\right)|x_{k}\right]-V\left(x_{k}\right)\\ \; & \;\leq -\lambda_{\min}\left(Q+\beta I\right){∥x_{k}∥}^{2}+\epsilon_{B}+\frac{M_{V}}{2}\mathrm{tr}\left(\Sigma_{\tilde{w}}\right). \end{array}$$

*Consequently, the conditional expected drift is negative whenever*

(25) $$∥x_{k}∥>r_{\beta }≜\sqrt{\frac{\epsilon_{B}+M_{V}\mathrm{tr}\left(\Sigma_{\tilde{w}}\right)/2}{\lambda_{\min}\left(Q+\beta I\right)}}.$$

**Proof.**  Let ${\bar{x}}_{k+1}=f\left(x_{k},\mu_{W^{*}}\left(x_{k}\right)\right)$. All expectations below are conditional on $x_{k}$.

*Step 1* (Nominal decrease). By Assumption 7 and $l_{r}\left(x,u\right)\geq \lambda_{\min}\left(Q+\beta I\right){∥x∥}^{2}$,

(26) $$\begin{array}{cc} V\left(x_{k}\right)-V\left({\bar{x}}_{k+1}\right)\; & \geq l_{r}\left(x_{k},\mu_{W^{*}}\left(x_{k}\right)\right)-\epsilon_{B}\\ \; & \geq \lambda_{\min}\left(Q+\beta I\right){∥x_{k}∥}^{2}-\epsilon_{B}. \end{array}$$

The second inequality follows from the quadratic lower bound of $l_{r}$ in (12), because $l_{r}\left(x,u\right)\geq x^{T}\left(Q+\beta I\right)x$.

*Step 2* (Disturbance increment). The disturbed successor is $x_{k+1}={\bar{x}}_{k+1}+{\tilde{w}}_{k}$. Given $x_{k}$, ${\bar{x}}_{k+1}$ is deterministic and randomness comes solely from ${\tilde{w}}_{k}$. A second-order Taylor expansion of *V* at ${\bar{x}}_{k+1}$ (Lagrange remainder form), along the segment specified in Assumption 8, gives

(27) $$V\left({\bar{x}}_{k+1}+{\tilde{w}}_{k}\right)\leq V\left({\bar{x}}_{k+1}\right)+\nabla V{\left({\bar{x}}_{k+1}\right)}^{T}{\tilde{w}}_{k}+\frac{M_{V}}{2}{∥{\tilde{w}}_{k}∥}^{2}.$$

Equivalently, the Lagrange remainder is evaluated at an intermediate point $\xi_{k}={\bar{x}}_{k+1}+t_{k}{\tilde{w}}_{k}$ with $t_{k}\in \left[0,1\right]$. The first-order term vanishes after conditioning because $\nabla V({\bar{x}}_{k+1})$ is deterministic given $x_{k}$ and $E[{\tilde{w}}_{k}|x_{k}]=0$. For the second-order term, $\xi_{k}$ depends on ${\tilde{w}}_{k}$, so $\nabla^{2}V\left(\xi_{k}\right)$ cannot be pulled outside the expectation. Assumptions 5 and 8 instead provide the pathwise bound

(28) $${\tilde{w}}_{k}^{T}\nabla^{2}V\left(\xi_{k}\right){\tilde{w}}_{k}\leq M_{V}{∥{\tilde{w}}_{k}∥}^{2}.$$

This is a pathwise inequality that does not rely on statistical independence between $\nabla^{2}V\left(\xi_{k}\right)$ and ${\tilde{w}}_{k}$. Taking the conditional expectation and using $E[∥{\tilde{w}}_{k}{∥}^{2}|x_{k}]=\mathrm{tr}\left(\Sigma_{\tilde{w}}\right)$ yields

(29) $$E\left[V\left(x_{k+1}\right)|x_{k}\right]-V\left({\bar{x}}_{k+1}\right)\leq \frac{M_{V}}{2}\mathrm{tr}\left(\Sigma_{\tilde{w}}\right).$$

The use of the Hessian bound $M_{V}$ requires $\xi_{k}\in D$. For a quadratic VFA, the Hessian bound holds globally and this requirement is automatically satisfied. For a higher-order VFA, the segment-containment condition in Assumption 8 ensures that every intermediate point remains in the stated domain.

*Step 3* (Combination). From the nominal decrease and the disturbance increment,

(30) $$\begin{array}{cc} \; & E\left[V\left(x_{k+1}\right)|x_{k}\right]-V\left(x_{k}\right)\\ \; & \;=\left[E\left[V\left(x_{k+1}\right)|x_{k}\right]-V\left({\bar{x}}_{k+1}\right)\right]\\ \; & \;-\left[V\left(x_{k}\right)-V\left({\bar{x}}_{k+1}\right)\right]\\ \; & \;\leq -\lambda_{\min}\left(Q+\beta I\right){∥x_{k}∥}^{2}+\epsilon_{B}+\frac{M_{V}}{2}\mathrm{tr}\left(\Sigma_{\tilde{w}}\right), \end{array}$$

which proves (24). When $∥x_{k}∥>r_{\beta }$, the right-hand side is strictly negative by (25). This establishes the stated conditional one-step result without asserting recursive feasibility, set invariance, or trajectory-level ultimate boundedness. □

**Remark 4** (Interpretation of β)**.**
*If $M_{V}$, $\Sigma_{\tilde{w}}$, and $\epsilon_{B}$ are held fixed, $r_{\beta }$ decreases as $\lambda_{\min}\left(Q+\beta I\right)$ increases. In the learned controller, however, V, $M_{V}$, and $\epsilon_{B}$ can themselves depend on β. Equation* (25) *is therefore a conditional sensitivity relation, not a proof that increasing β universally improves a closed-loop stability margin. When $\Sigma_{\tilde{w}}=0$ and $\epsilon_{B}=0$,* (24) *recovers a nominal decrease inequality; an asymptotic-stability conclusion still requires the usual feasibility and invariance conditions.*

**Remark 5** (Scope)**.**
*Assumption 8 is not a recursive-feasibility proof. The β term changes the cost but not the constraint sets, and the absence of infeasibility in the reported simulations is empirical rather than a guarantee. The theorem also does not apply to the untruncated Gaussian simulation disturbance without a separate tail and trajectory analysis.*

## 4. Results and Discussion

### 4.1. Simulation Setup

Simulations are conducted on a second-order linear system and a nonlinear nonholonomic vehicle system; the latter captures the kinematics of a differential-drive mobile robot. The linear system admits an analytical linear-quadratic regulator (LQR) solution and serves to verify RL-MPC convergence behavior; the nonlinear system introduces Gaussian disturbances and model mismatch to examine robustness. Baseline parameters for both systems follow existing RL-MPC settings for direct comparability with prior benchmarks [33]. The simulator directly supplies the state vector to the controller. No sensor model, measurement noise, state estimator, or sensor-fusion algorithm is included, so the results evaluate the control layer rather than a complete sensing-to-control stack.

Consider the discrete-time linear system $x_{k+1}=Ax_{k}+Bu_{k}$ with $x_{k}\in R^{2}$, $u_{k}\in R$, and

(31) $$A=\left[\begin{array}{cc} 1 & 0.5\\ 0.1 & 0.5 \end{array}\right],\;B=\left[\begin{array}{c} 1\\ 0 \end{array}\right]$$

The cost weights are $Q=I_{2}$, R=0.5, yielding an LQR optimal cost of $J^{*}\approx 3.49$. The RL-MPC prediction horizon is N=3 with a polynomial basis of order p=2. Four methods are compared on the linear system: RLMPC, LQR, MPC without terminal cost (N=3), and MPC with terminal cost (N=3), where LQR serves as the global optimum benchmark.

Consider the nonholonomic kinematic vehicle model with additive disturbances:

(32) $$x_{k+1}=x_{k}+g\left(x_{k}\right)u_{k}+w_{k}^{G}$$

The state $x_{k}={\left[\chi_{k},y_{k},\theta_{k}\right]}^{T}$ contains the planar coordinates and heading angle, and the control input $u_{k}={\left[v_{k},\omega_{k}\right]}^{T}$ consists of the linear and angular velocities. The system matrix

(33) $$g\left(x_{k}\right)=\delta \left[\begin{array}{cc} \cos\theta_{k} & 0\\ \sin\theta_{k} & 0\\ 0 & 1 \end{array}\right]$$

encodes the nonholonomic coupling between displacement and heading, with sampling interval δ=0.2s. The disturbance $w_{k}^{G}∼N\left(0,\sigma^{2}I\right)$ is additive Gaussian white noise whose intensity σ varies by scenario. Input constraints are $\left|v_{k}\right|\leq 1\;m/s$ and $\left|\omega_{k}\right|\leq 4\;\mathrm{rad}/s$; the initial state is $x_{0}={\left[1.5,1.5,\pi /4\right]}^{T}$. The initial planar displacement is $\sqrt{1.5^{2}+1.5^{2}}\approx 2.12\;m$, so the maneuver is explicitly meter-scale. For comparison, Cao et al. use a 0.4m/s limit for nonholonomic differential-steering path following, whereas Ou et al. use a 1m/s limit for indoor mobile-robot navigation [3,4]. The setup is therefore best interpreted as a simulation-level, low-to-moderate-speed indoor mobile-robot maneuver with differential-drive kinematics, rather than as a parameterization of a specific hardware platform. The control objective is to drive the vehicle to the neighborhood of the origin.

Five methods are compared on the nonlinear system: (i) VR-RLMPC (proposed), using the robust cost $l_{r}=x^{T}\left(Q+\beta I\right)x+u^{T}Ru$ with β=0.5 and N=5; (ii) RLMPC, the original scheme with β=0; (iii) long-horizon MPC with N=20 and no terminal cost; (iv) MPC without terminal cost, N=5; (v) MPC with terminal cost, N=5, where the terminal cost is obtained from DARE at the linearized equilibrium. The value β=0.5 is chosen on the same order as $\lambda_{\min}\left(Q\right)$ so that the regularization intensity is comparable to the nominal state weight without excessively distorting the learning target.

The nominal nonlinear configuration uses Q=diag(1,1,0.2), R=diag(0.05,0.01), and Gaussian disturbance level σ=0.05 when enabled; activation follows the batch-specific training and evaluation protocols stated below. The lower heading weight prioritizes position tracking, while the lower angular-velocity penalty permits flexible heading adjustment. The value function uses a polynomial basis of order p=4. Simulations run in MATLAB R2025a. The fmincon solver uses SQP first, with interior-point or active-set methods as fallbacks after a failed attempt. RLMPC and VR-RLMPC use the same learning safeguards from Section 3.3. The proposed β term changes the cost matrix without changing the online decision variables, constraints, or sampling loops. The archived ACT values report average solver time per MPC step.

Closed-loop performance is measured by the accumulated cost (ACC):

(34) $$\mathrm{ACC}=\sum_{k=0}^{T-1}l\left(x_{k},u_{k}\right)$$

For cross-method comparability, ACC is computed using the nominal cost *l* (not $l_{r}$). The reported values are means $\bar{\mathrm{ACC}}$ over multiple independent runs. VFA weight-update settling is summarized by an empirical convergence-step index, denoted CR. For each run, let $d_{j}={∥W_{j+1}-W_{j}∥}_{2}$ over the learning window and define the run-specific relative threshold $\epsilon_{\mathrm{rel}}=0.15\max_{j}d_{j}$. Then

(35) $$\mathrm{CR}=\inf\{j\in N|d_{j+i}<\epsilon_{\mathrm{rel}},\;i=0,\ldots ,4\}.$$

CR is undefined if no five-update window meets this criterion. Because the threshold is normalized by each run’s peak update norm, uniform rescaling of all update magnitudes alone does not automatically reduce CR. Nevertheless, CR remains a relative settling indicator; a finite CR does not prove finite-sample convergence, weight boundedness without safeguards, or resolution of the exploration–exploitation trade-off.

The following derived metrics are also used. The performance degradation rate (PDR) quantifies the effect of model mismatch on a single method:

(36) $$\mathrm{PDR}\left(\kappa \right)=\frac{\mathrm{ACC}(\kappa )-\mathrm{ACC}(1)}{\mathrm{ACC}(1)}\times 100\%$$

The ACC improvement rate measures the cost reduction of VR-RLMPC relative to RLMPC:

(37) $$\mathrm{ACC}\;\mathrm{imp}.=\frac{{\mathrm{ACC}}_{\mathrm{RLMPC}}-{\mathrm{ACC}}_{\mathrm{VR}\text{-}\mathrm{RLMPC}}}{{\mathrm{ACC}}_{\mathrm{RLMPC}}}\times 100\%$$

The VFA terminal cost ratio (VTCR) measures the cost reduction of VR-RLMPC relative to MPC with terminal cost:

(38) $$\mathrm{VTCR}=\frac{{\mathrm{ACC}}_{\mathrm{MPC}+\mathrm{TC}}-{\mathrm{ACC}}_{\mathrm{VR-RLMPC}}}{{\mathrm{ACC}}_{\mathrm{MPC}+\mathrm{TC}}}\times 100\%$$

The ACC variance reduction (AVR) quantifies the compression of ACC standard deviation by β:

(39) $$\mathrm{AVR}=\frac{{\mathrm{std}}_{\mathrm{RLMPC}}-{\mathrm{std}}_{\mathrm{VR-RLMPC}}}{{\mathrm{std}}_{\mathrm{RLMPC}}}\times 100\%$$

### 4.2. Linear System Verification

The LQR analytical solution serves as a quantitative benchmark. Simulations are conducted under both noise-free (σ=0) and noisy (σ=0.05) conditions. The noise-free case is deterministic and each method is run once from identical initial conditions; the noisy case is repeated 10 times independently, reporting means and standard deviations.

**Figure 2:**
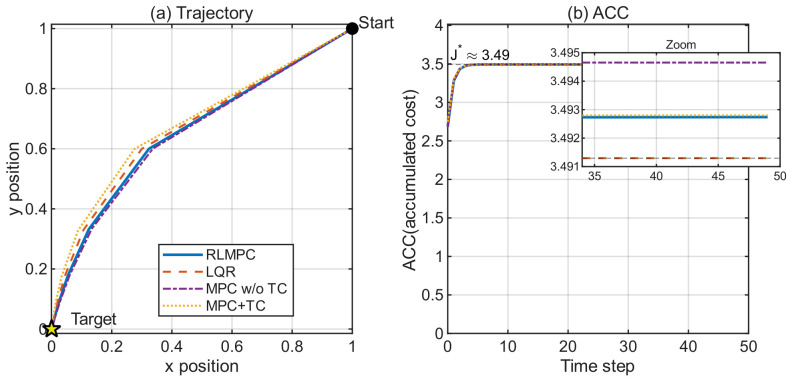
Linear system verification: phase-plane trajectories and ACC curves.

Figure 2 shows the state trajectories and ACC curves for the four methods under the noise-free condition. The four trajectories nearly coincide, and all ACC values converge to the analytical optimum: RLMPC yields 3.493, LQR 3.491, MPC without terminal cost 3.495, and MPC with terminal cost 3.493, with a maximum relative deviation below 0.2%. After introducing noise, the ACC of all four methods increases synchronously to approximately 3.86±0.11, and the difference between RLMPC and LQR does not exceed 0.001%. The near-identical performance indicates that the learned terminal cost reproduces LQR-level performance on the tested linear system.

### 4.3. Nonlinear Vehicle System: Nominal Configuration

This subsection examines the practical effect of β compensation on the nonlinear vehicle system under Gaussian disturbances. Under the nominal parameter configuration (σ=0.05, κ=1, no model mismatch), five methods are compared using 7 independent runs per method.

**Figure 3:**
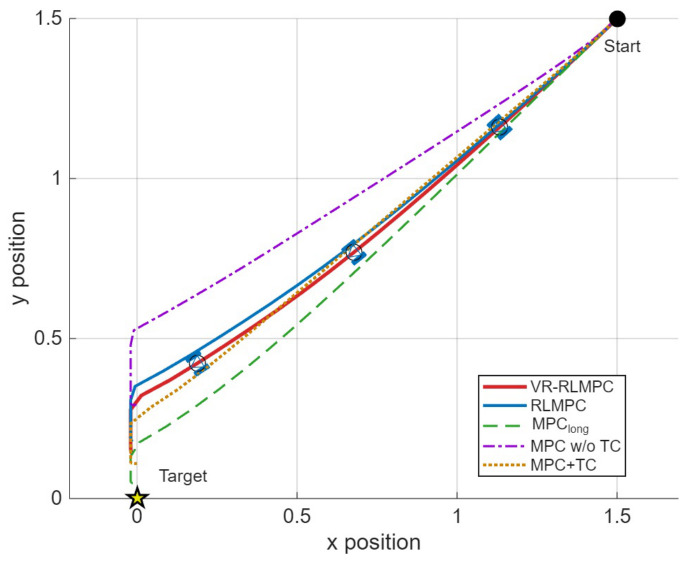
XY trajectory comparison on the nonlinear vehicle system.

Figure 3 shows the XY trajectories of the five methods. VR-RLMPC and RLMPC follow similar paths, both tracing a compact arc from the initial point toward the origin; MPC with terminal cost produces a comparable trajectory. MPC without terminal cost, lacking long-horizon cost information, shows a clear deviation with a lag in the *y* direction. Long-horizon MPC reaches the target vicinity but exhibits a larger path envelope.

**Figure 4:**
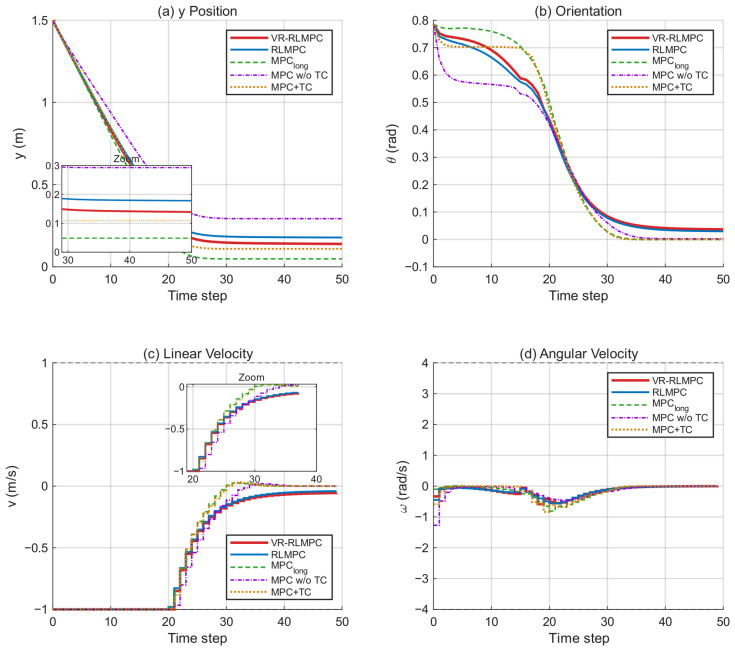
State and control responses under the nominal configuration (gray dashed lines: numerical input bounds).

Figure 4 further shows the time evolution of state components and control inputs. Differences are most pronounced in the *y* position and heading angle θ: the *y* component of MPC without terminal cost converges most slowly, and θ remains elevated for an extended period. Regarding control inputs, both VR-RLMPC and RLMPC reach the lower linear velocity constraint (v=−1m/s) during the first 20 steps, then relax smoothly to zero.

Table 1 lists the ACC statistics under the nominal configuration. The five primary methods use 7 independent runs, whereas the separately evaluated ${\mathrm{MPC}}_{Q_{r}}$ baseline uses 3 independent runs.

VR-RLMPC reduces ACC by approximately 0.7% in the 7-run nominal comparison and by 1.36% in the separate 3-run stochastic-TD comparison. These changes are modest relative to run-to-run dispersion; the clearer nominal effect is the approximately 27% lower empirical CR settling-step index. The mismatch sweep and high-demand configuration produce larger, single-digit gains. Long-horizon MPC with N=20 achieves slightly lower ACC than the N=5 RLMPC methods. MPC without terminal cost exceeds VR-RLMPC by 6.6%, consistent with its trajectory lag. MPC with terminal cost is nearly tied with VR-RLMPC (42.52 vs. 42.53), indicating comparable nominal performance from the learned VFA and DARE terminal costs.

To disentangle the source of β compensation (whether from the stage cost modification itself or from VFA online learning), an ${\mathrm{MPC}}_{Q_{r}}$ baseline is introduced: MPC with the modified cost matrix Q+βI but without value function learning (N=5, no terminal cost). As shown in Table 1, ${\mathrm{MPC}}_{Q_{r}}$ achieves an ACC of 50.18±1.26, exceeding VR-RLMPC by 18.0% (50.18 vs. 42.53) and performing worse than MPC without terminal cost (45.34). In this tested setting, modifying the stage cost matrix alone does not substitute for the VFA terminal cost; the increased stage cost magnitude from βI degrades short-horizon MPC performance. This baseline supports the interpretation that the observed advantage depends on the interaction between the modified stage cost and online VFA learning, rather than on the state-weight change alone. The mechanism is therefore framed as learning-side regularization rather than simple MPC weight scaling.

**Figure 5:**
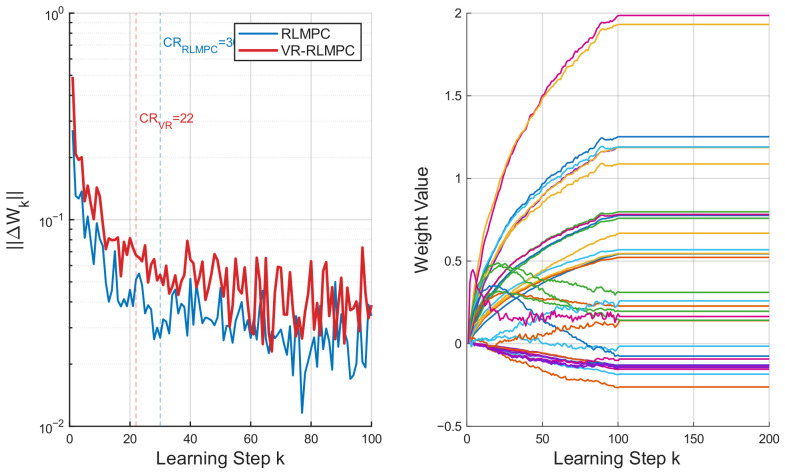
VFA weight-update histories under the nominal configuration. In the left panel, blue denotes RLMPC and red denotes VR-RLMPC; in the right panel, the colored lines distinguish the individual VR-RLMPC weight components.

Figure 5 compares the weight update processes of RLMPC (β=0) and VR-RLMPC (β=0.5). The left panel shows $∥\Delta W_{k}∥$ versus learning step: VR-RLMPC drops below the relative five-update settling threshold at approximately step 22 (CR=22), while RLMPC requires about 30 steps (CR=30), giving an observed CR reduction of approximately 27%. The right panel displays the evolution of individual VR-RLMPC weight components over the 100-step learning window; it is descriptive and does not establish finite-sample convergence outside this run.

An alternative to β regularization is to perform Monte Carlo averaging of the terminal value during TD learning, explicitly accounting for the disturbance effect on value function estimation. Specifically, the TD target terminal value $W^{T}\phi \left(x_{N|k}\right)$ is replaced by

(40) $$\begin{array}{c} {\hat{V}}_{\mathrm{MC}}\left(x_{N|k};W\right)=\frac{1}{M}\sum_{m=1}^{M}W^{T}\phi \;\left(x_{N|k}+w^{\left(m\right)}\right),\;\;\;\;\;\;\;\;\;\;\;\;\;\;\;\;\;\;\;\;\;\;\;\;\;\;\;\;\;\;\;\;\;\;\;\;\;\;\;\;\;\;\;\;\;\;\;\;\;\;\\ \;\;\;\;\;\;\;\;\;\;\;\;\;\;\;\;\;\;\;\;\;\;\;\;\;\;\;\;\;\;\;\;\;\;\;\;\;\;\;\;\;\;\;\;\;\;\;\;\;\;w^{\left(m\right)}\overset{i.i.d.}{∼}N\left(0,\sigma^{2}I\right) \end{array}$$

This method approximates $E_{w}\left[\hat{V}\left(x_{N|k}+w\right)\right]$ via sampling, with computational overhead growing linearly in *M* (*M* additional ϕ(·) evaluations per step). For a fair comparison with the implicit regularization of VR-RLMPC, all Stochastic TD variants use the nominal cost *l* (β=0) and handle disturbances solely through terminal value averaging. With M∈{5,10,20}, alongside RLMPC (β=0) and VR-RLMPC (β=0.5), 3 independent runs are performed under the nominal configuration; results are listed in Table 2.

The three Stochastic TD variants improve ACC by no more than 0.5%, with no monotone improvement as *M* increases from 5 to 20. VR-RLMPC achieves an improvement of +1.36%, compared with +0.41% for the best Stochastic TD variant (M=10), and obtains the lowest ACC across all 3 random seeds (paired win rate 3/3). These absolute ACC differences remain modest. Regarding empirical VFA weight-update settling, VR-RLMPC achieves CR=27.7, a reduction of approximately 27% relative to RLMPC, while the Stochastic TD variants have CR=36.7–41.7 in this nominal comparison. At σ=0.05, $E\left[\phi \left(x+w\right)\right]\approx \phi \left(x\right)+O\left(\sigma^{2}\right)$, so Monte Carlo averaging corrects only a second-order perturbation of the terminal value estimate. In contrast, β regularization directly changes the stage-cost matrix through Q→Q+βI. This distinction provides a mechanism-level interpretation of the different CR values observed in the tested nominal configuration, not a convergence-rate proof.

Under the nominal configuration, the primary observed benefit of β is the approximately 27% lower empirical CR settling-step index; the absolute ACC gain remains modest. In this regime, RLMPC already operates near optimality, constraining the ACC improvement from β. The next subsection increases task difficulty to examine whether the ACC improvement changes in the tested configurations.

### 4.4. Parameter Sensitivity and Model-Mismatch Analysis

The nominal-configuration simulations show that β improves ACC by approximately 0.7% in the 7-run comparison, with the larger observed difference appearing in CR (27%). The following analysis tests whether the ACC improvement changes when the simulation places greater weight on heading accuracy (large $Q_{\theta }$) or control effort (large *R*). Single-factor ablation identifies the tested settings in which the observed β benefit is larger, after which the two dominant factors are combined into a high-demand simulation configuration.

#### 4.4.1. Single-Factor Ablation

Four factors that may affect terminal-cost accuracy requirements are selected. Factor A (tightened linear velocity, $\left|v_{k}\right|\leq 0.5$) compresses the constraint feasible region; B (increased $Q_{\theta }$, with $Q_{\theta }=1,2,3$) raises the state-cost weight along the heading dimension; C (increased control cost, R×2) strengthens the penalty on control effort; and D (increased initial offset) extends the transient process. Only one factor is varied at a time, with σ=0.05 and κ=1. Increasing $Q_{\theta }$ is the dominant tested factor: B2 ($Q_{\theta }=2$) achieves an improvement of +3.72%, compared with nominal (+1.12%), A (+0.75%), and C (+1.16%). The full ablation also includes B1 ($Q_{\theta }=1$, +2.35%), B3 ($Q_{\theta }=3$, +3.96%), and D (+0.57%); the $Q_{\theta }$ results increase monotonically over the three tested values.

Increasing $Q_{\theta }$ requires the value function $\hat{V}$ to represent a more strongly weighted heading dimension. The β compensation via Q+βI changes the learning signal across all state dimensions, with the largest observed difference under high-$Q_{\theta }$ settings. VR-RLMPC also achieves lower ACC than MPC with terminal cost in both B2 and B3: 57.18 vs. 59.03 in B2 (3.2% lower), and 64.08 vs. 68.10 in B3 (6.3% lower). These results indicate better observed performance than the DARE linearized terminal-cost baseline in the tested high-$Q_{\theta }$ tasks.

#### 4.4.2. High-Demand Engineering Configuration (B2+C): Statistical Verification

**Figure 6:**
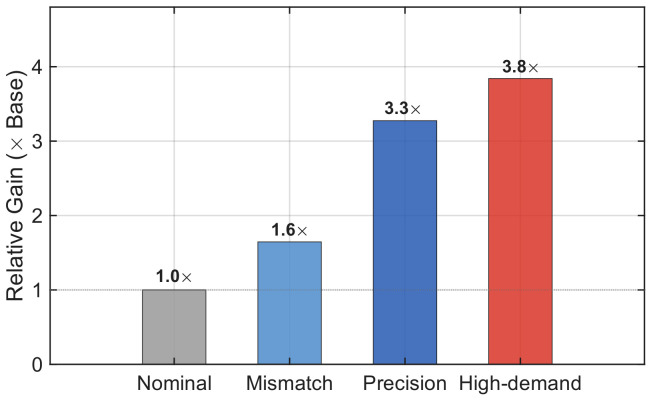
Relative ACC improvement with *β* regularization across four representative configurations. The bar colors distinguish the four labeled configurations and do not encode an additional quantitative variable.

Figure 6 provides a coarse cross-configuration overview. Mismatch denotes the κ=0.5 model-mismatch result, Precision denotes B2, and High-demand denotes B2+C. The exact configuration-specific percentages and the detailed A/B/C/D single-factor results are reported in the text; the bars are descriptive rather than a matched statistical comparison.

The ablation results show that A and D have smaller effects on β improvement (+0.75%/+0.57%), while the tested B settings produce larger changes. B2+C combines a larger heading weight with a larger control-effort weight. Specifically, B2 ($Q_{\theta }=2$) is selected as the primary configuration: the improvement rate is near saturation ($Q_{\theta }$ from 2 to 3 adds only 0.24 percentage points). On this basis, C (R×2) is added. The final high-demand configuration (denoted B2+C) uses Q=diag(1,1,2), R=diag(0.1,0.05), with all other parameters unchanged. Across 5 independent repeats, RLMPC (β=0) achieves an ACC of 63.03±0.07, whereas VR-RLMPC achieves an ACC of 60.28±0.05, an improvement of +4.36% (one-sided paired *t*-test, $p=1.31\times 10^{-7}$). Compared with MPC with terminal cost (ACC = 62.40), VR-RLMPC is lower by 3.4% under this high-$Q_{\theta }$ task.

The B2+C improvement is statistically significant in the one-sided paired test ($p=1.31\times 10^{-7}$). This *p*-value reflects the high reproducibility of the mean difference between the two methods under low-variance conditions, not the engineering magnitude of the absolute improvement. The ACC standard deviation in B2+C (0.05–0.07) is far smaller than that of the nominal configuration (≈3.4). The exact 4.36% high-demand and 1.12% nominal single-factor values, rather than the coarse overview in Figure 6, are used for quantitative comparison. This scenario-specific result combines a single-digit effect with unusually low within-batch variance, whose source was not isolated.

#### 4.4.3. Model-Mismatch Robustness

In practical systems, the prediction model typically deviates from the true dynamics. A mismatch factor κ is introduced: the MPC prediction model uses sampling interval κδ (the true system uses δ=0.2s); κ≠1 indicates model overestimation (κ>1) or underestimation (κ<1) of the time step. The sweep uses κ∈{0.5,0.7,1.0,1.5,2.0} with 7 independent runs per group under the nominal parameter configuration.

**Figure 7:**
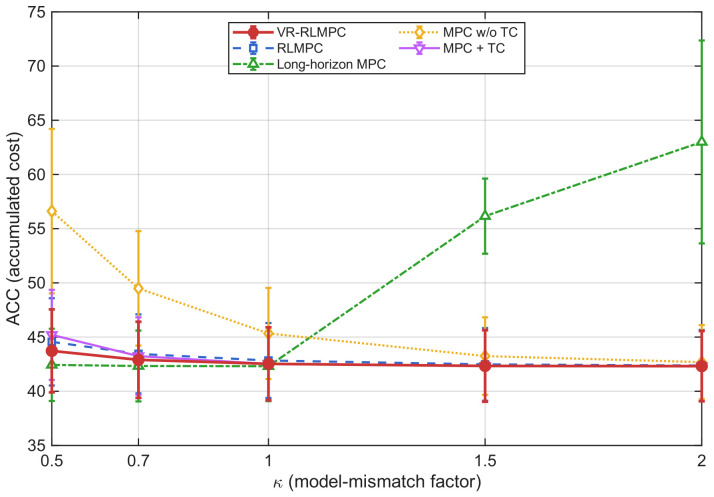
Mean ACC under model-mismatch factor *κ* (error bars: ±1 standard deviation).

Figure 7 and Table 3 summarize the ACC of each method across different κ values.

The data reveal two opposing degradation patterns. When κ<1, the model underestimates the sampling interval; MPC plans aggressively, and the true system response overshoots the prediction. MPC without terminal cost degrades first: at κ=0.5, ACC rises to 56.62 (+25% over baseline), and the standard deviation increases to 7.59. When κ>1, the model overestimates the sampling interval and prediction errors accumulate along the longer horizon. Long-horizon MPC then degrades first: at κ=2.0, ACC increases to 63.00 (+49%), with a standard deviation of 9.36. The two degradation directions are complementary: the mismatch direction determines which prediction structure is impaired first.

The RLMPC family shows little ACC variation across the tested κ values. VR-RLMPC ACC ranges from 42.32 to 43.73, with a maximum deviation of 2.8%; the standard deviation stays within 3.24–3.83. The short prediction horizon (N=5) limits prediction error accumulation, and the VFA terminal cost provides a consistent long-horizon cost estimate in this sweep.

Regarding empirical VFA weight-update settling (lower half of Table 3), VR-RLMPC has lower CR than RLMPC at all five κ levels, with reductions of 29.5% (κ=0.5), 24.7% (κ=0.7), 23.8% (κ=1.0), 24.5% (κ=1.5), and 23.3% (κ=2.0), averaging approximately 25%. At κ=0.5 the absolute CR reduction is largest (46.4 → 32.7, −13.7 steps), coinciding with the largest ACC improvement from β in this sweep; this empirical association is not a finite-sample convergence-rate result.

From the β compensation perspective, the improvement magnitude follows a monotone trend with mismatch direction. VR-RLMPC improves ACC over RLMPC by +1.9% at κ=0.5, +1.3% at κ=0.7, +0.7% at κ=1.0, and +0.1% at κ=2.0. When κ<1, model underestimation amplifies the effective disturbance, and the largest observed benefit of β occurs in this regime. For κ>1, the smaller improvement is consistent with prediction-error accumulation becoming more important relative to VFA accuracy.

**Figure 8:**
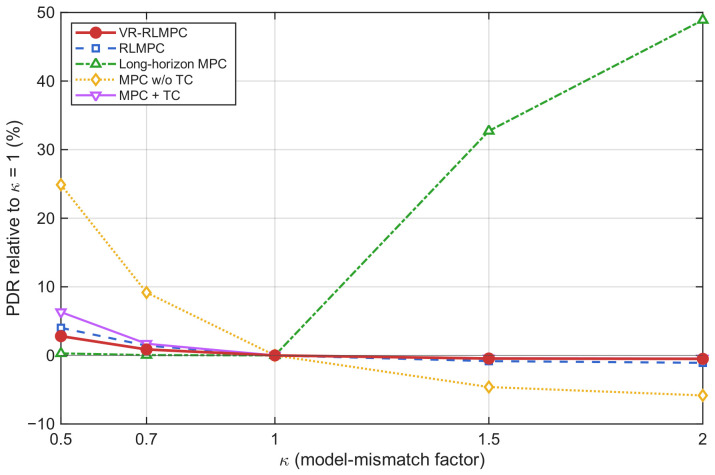
Performance degradation rate (PDR) as a function of *κ*.

Figure 8 shows the PDR (defined in Section 4.1) as a function of κ. The maximum |PDR| of VR-RLMPC is 2.8% (κ=0.5); RLMPC reaches 4.0%, and MPC with terminal cost reaches 6.3%. Long-horizon MPC and MPC without terminal cost have maximum PDR values of 48.9% and 24.9%, respectively.

**Figure 9:**
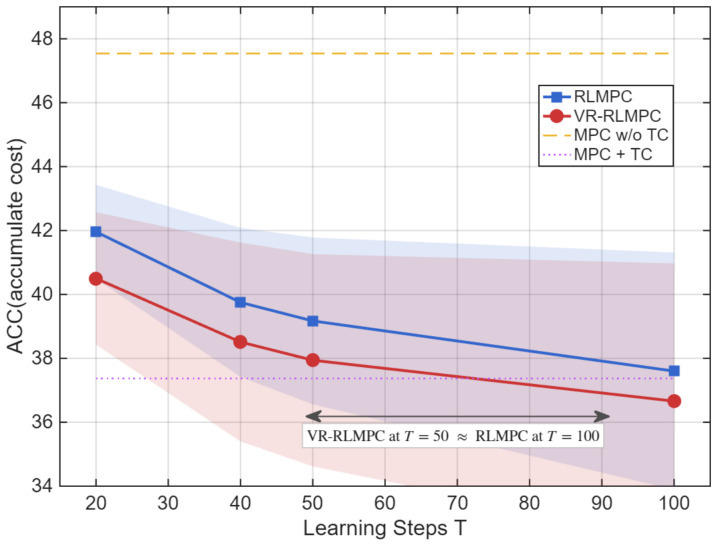
Training-budget comparison at *κ* = 0.5 (shaded region: ±1*σ*). Colors distinguish the methods identified in the legend.

At κ=0.5, VFA learning contributes most to final performance. To assess performance under a limited training budget, the learning phase length *T* is reduced from 100 to 20. Both β=0 and β=0.5 are run 3 times at each budget. Figure 9 shows ACC versus *T*. At T=50, VR-RLMPC achieves an ACC of 37.94±3.32, close to the RLMPC value of 37.60±3.71 at T=100 (a difference of 0.9%). In this simulated setting, β compensation therefore reduces the number of learning steps needed to reach a comparable ACC level. The paired comparison shows that β=0.5 outperforms β=0 at all 4 training-budget levels (a 100% win rate across 12 pairs). The advantage increases monotonically as the training budget decreases, from ΔACC=2.5% at T=100 to 3.5% at T=20. This result should be interpreted as a simulated training-budget effect rather than a general claim of RL sample efficiency, and it requires future validation with a physical sensing and estimation chain.

**Figure 10:**
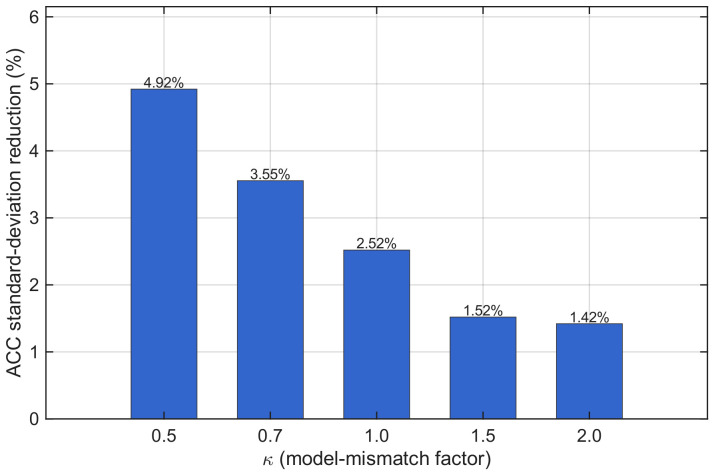
ACC standard-deviation reduction (AVR) under model mismatch.

Figure 10 provides a descriptive comparison of dispersion within the same 7-run model-mismatch sweep. The ACC standard deviation of VR-RLMPC is lower than that of RLMPC at all five tested κ values; the corresponding AVR descriptor decreases from 4.9% at κ=0.5 to 1.4% at κ=2.0. These values summarize dispersion in the tested runs and are not a probabilistic safety, stability, or tail-risk guarantee.

### 4.5. β Sensitivity Analysis

**Figure 11:**
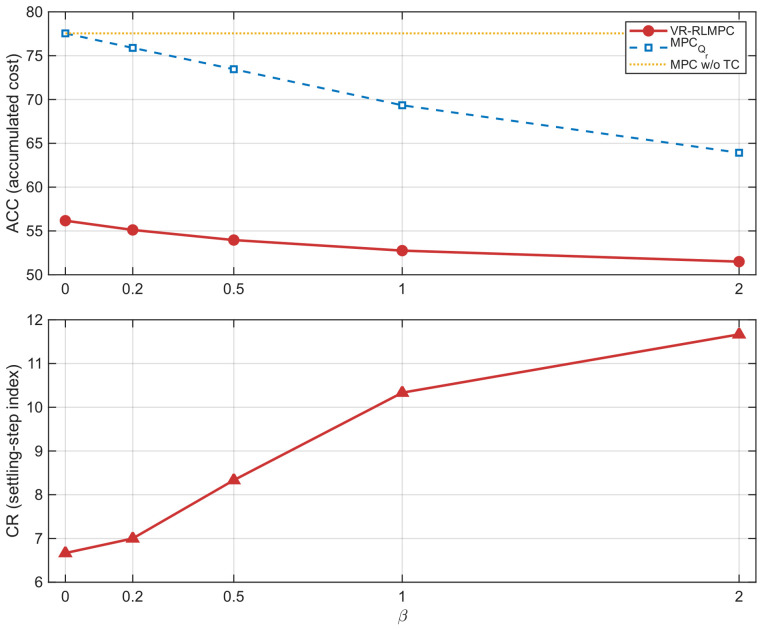
Mean ACC and CR as functions of *β*.

All preceding simulations use β=0.5. This subsection sweeps β∈{0,0.2,0.5,1.0,2.0} under the high-demand configuration (B2+C) with 3 independent repeats (paired design) to examine the effect of β on performance and empirical VFA weight-update settling. Gaussian disturbances (σ=0.05) are applied during both training and evaluation for every β value. Figure 11 shows the results.

ACC decreases monotonically with β (i.e., performance improves monotonically), from 56.17±1.30 at β=0 to 51.50±2.37 at β=2.0 (improvement of 8.3%), with no degradation inflection point within the tested range. Simultaneously, CR increases monotonically from 6.7±1.2 to 11.7±4.7. An ACC–CR trade-off therefore exists: β=0.5 captures approximately 47% of the maximum ACC improvement (+3.9%) while increasing CR by 24%; β=2.0 achieves the best ACC but increases CR by 75%. Within the tested range, β=0.5 provides an engineering compromise between ACC and CR. All 15 archived runs yield finite ACC and CR records under the reported safeguards. Untested β–α combinations and more dynamic regimes remain outside this single-configuration sweep.

Equation (25) provides a complementary assumption-conditioned interpretation: if the Bellman-residual bound, Hessian bound, and disturbance covariance are held fixed, increasing β raises $\lambda_{\min}\left(Q+\beta I\right)$ and reduces the radius $r_{\beta }$ outside which the conditional one-step drift upper bound is negative. This coefficient-level relation is not a formal stability margin, and neither it nor the single-configuration sweep constitutes a multi-configuration transient-response analysis.

At the same β, VR-RLMPC consistently outperforms the pure cost-modification baseline MPC (Q+βI, no VFA) in the tested sweep: at β=0.5 the gap reaches 26.6% (53.96 vs. 73.46). This comparison supports the interpretation that the observed improvement depends on the interaction between regularization and online VFA learning, rather than on increasing the stage-cost weight alone.

To provide an implementation-level timing reference, the archived simulation outputs report ACT, defined as the average solver time per MPC step. Table 4 summarizes representative ACT values.

VR-RLMPC has slightly higher average solver time than RLMPC in these runs. All representative mean ACT values remain below the 0.2 s simulation sampling interval, and β adds no online decision variables, constraints, or sampling loops. This is encouraging for transfer, but ACT is an average solver-timing measurement rather than a worst-case end-to-end latency. Embedded real-time feasibility requires implementation on a target processor, integration with the sensing and state-estimation stack, and platform-specific worst-case timing tests.

### 4.6. Scope and Limitations

The results establish the behavior of the proposed controller in simulation, not the performance of a complete autonomous mobile robot. The simulated controller receives the state directly, whereas a physical platform would obtain feedback through onboard sensors, calibration, filtering, and state estimation. Measurement noise, estimator delay, state-estimation error, sensor faults, and multi-sensor fusion can alter the effective closed-loop disturbance and are not represented by the additive process disturbance used here. The theoretical result is also conditional on the stated VFA, feasibility, domain-containment, and bounded-support assumptions; recursive feasibility and a trajectory-level guarantee for untruncated Gaussian disturbances remain open. Accordingly, the conclusions are limited to the state-feedback simulations and the conditional analytical model; sensor-integrated validation is required before extending them to a complete autonomous mobile robot.

The evaluation does not cover heavy-tailed or biased disturbances, hardware-in-the-loop operation, or physical robot tests. Actuator saturation, backlash, wheel slip, communication delay, and estimator–controller coupling may require explicit robust constraints or safety filters. The isotropic regularization term adds no direction-specific priority beyond *Q*, *R*, *f*, X, and U; anisotropic regularization is a natural extension. The finite β sweep also leaves high-gain updates and untested β–α combinations for future study.

The numerical comparison covers the implemented RLMPC, MPC, LQR, ${\mathrm{MPC}}_{Q_{r}}$, and stochastic-TD baselines. Robust MPC and DRL–MPC studies with different uncertainty models, learned objects, and evaluation protocols are used for literature positioning; matched numerical comparison requires separate implementations.

## 5. Conclusions

This work introduces isotropic state regularization into the reinforcement learning-based model predictive control (RL-MPC) framework [33], yielding value-regularized RLMPC (VR-RLMPC). By inserting the same β term into the physical state penalty and the *N*-step TD target, VR-RLMPC couples the control and learning objectives without adding online MPC variables, constraints, or sampling operations. The ${\mathrm{MPC}}_{Q_{r}}$ ablation indicates that changing the stage-cost matrix alone does not reproduce the learned-terminal-value result. Under explicit Bellman-residual, feasibility, domain-containment, and bounded-disturbance assumptions, the analysis gives a conditional one-step expected Lyapunov-drift bound. Simulations on a nonlinear nonholonomic vehicle system show lower empirical CR in the nominal and model-mismatch comparisons at β=0.5, while the sensitivity sweep identifies an ACC–CR trade-off. Nominal ACC reductions remain modest, and the larger high-demand improvement is scenario-specific and single-digit.

These findings are limited to the stated analytical assumptions and state-feedback simulations. They do not establish recursive feasibility, trajectory-level guarantees for untruncated Gaussian disturbances, embedded worst-case timing, or sensor-integrated and physical-robot performance. Future work will investigate trajectory-level probabilistic bounds, finite-sample VFA behavior, joint β–learning-rate design, and adaptive or anisotropic regularization. It will also integrate onboard sensing and state estimation, evaluate platform-specific embedded timing, and validate the controller on a physical unmanned mobile-robot testbed.

## Figures and Tables

**Table 1:** Accumulated cost comparison on the nonlinear vehicle system under the nominal configuration.

Method	ACC
VR-RLMPC	42.53±3.37
RLMPC	42.84±3.46
Long-horizon MPC	42.31±3.23
MPC w/o terminal cost	45.34±4.20
MPC w/ terminal cost	42.52±3.37
${\mathrm{MPC}}_{Q_{r}}$ (no VFA)	50.18±1.26

**Table 2:** Explicit stochastic temporal-difference (TD) baselines under the nominal configuration. ACC: accumulated cost; CR: empirical VFA settling-step index; ΔACC: percentage ACC improvement relative to RLMPC (positive values indicate lower ACC).

Method	ACC (Mean ± Std)	CR	ΔACC
RLMPC (*β* = 0)	35.54±4.88	38.3	—
VR-RLMPC (*β* = 0.5)	35.06±5.35	27.7	+1.36%
Stochastic TD (M=5)	35.43±5.06	39.3	+0.32%
Stochastic TD (M=10)	35.40±5.10	36.7	+0.41%
Stochastic TD (M=20)	35.42±5.07	41.7	+0.35%

ΔACC: improvement over RLMPC (β = 0); positive is better.

**Table 3:** Model-mismatch comparison. ACC is reported as mean ± standard deviation, whereas CR is the mean empirical VFA settling-step index.

***ACC (Accumulated Cost)***					
***κ***	**VR-RLMPC**	**RLMPC**	**Long-horizon MPC**	**MPC w/o TC**	**MPC w/ TC**
0.5	43.73±3.83	44.56±4.03	42.44±3.33	56.62±7.59	45.21±4.16
0.7	42.90±3.51	43.45±3.64	42.33±3.27	49.49±5.28	43.24±3.58
1.0	42.53±3.37	42.84±3.46	42.31±3.23	45.34±4.20	42.52±3.37
1.5	42.34±3.30	42.48±3.35	56.16±3.47	43.25±3.58	42.34±3.27
2.0	42.32±3.24	42.37±3.29	63.00±9.36	42.69±3.42	42.33±3.23
***CR (Empirical VFA Settling-Step Index)***					
***κ***	**VR-RLMPC**	**RLMPC**	**—**		
0.5	32.7	46.4			
0.7	29.3	38.9			
1.0	22.4	29.4			
1.5	20.9	27.7			
2.0	20.7	27.0			

**Table 4:** Solver timing across representative evaluation scenarios (ACT, seconds per MPC step).

Scenario	RLMPC ACT (s)	VR-RLMPC ACT (s)
Model mismatch	0.0173–0.0211	0.0241–0.0256
High-demand scenario	0.03997 ± 0.00081	0.04515 ± 0.00184
Limited training budget	0.0459–0.0519	0.0524–0.0605

Mismatch and training-budget entries are ranges across tested *κ* and *T* settings.

## Data Availability

The minimal dataset supporting the central findings of this study is provided as Supplementary Material. It contains raw and processed simulation results, metadata, and a MATLAB verification script. The complete source code is not publicly available.

## Supplementary Materials

The following supporting information can be downloaded at: https://www.mdpi.com/article/10.3390/s26144620/s1, Supplementary File S1: Minimal dataset supporting the reported simulation results, including raw and processed data, metadata, and MATLAB scripts for regenerating the processed summaries and verifying the central numerical claims.
